# Acute psilocybin or ethocybin leads to a sustained increase in Pavlovian aversion memory in male mice

**DOI:** 10.3389/fphar.2026.1918182

**Published:** 2026-09-22

**Authors:** David Ernst Hogg, Jennifer Romanos, Jamie Albiez, Céline Hofer, Sarah Wicki, Ali Özgür Argunşah, Hannes Sigrist, Jan Thomann, Dino Luethi, Franz X. Vollenweider, Christopher R. Pryce

**Affiliations:** 1 Preclinical Laboratory, Adult Psychiatry and Psychotherapy, University Hospital of Psychiatry and University of Zurich, Zurich, Switzerland; 2 Zurich Neuroscience Center, University of Zurich and ETH Zurich, Zurich, Switzerland; 3 Laboratory for Neural Circuit Assembly, Brain Research Institute, University of Zurich, Zurich, Switzerland; 4 Division of Clinical Pharmacology and Toxicology, Department of Pharmaceutical Sciences, University of Basel, Basel, Switzerland; 5 Division of Clinical Pharmacology and Toxicology, Department of Biomedicine, University Hospital Basel, Basel, Switzerland; 6 Adult Psychiatry and Psychotherapy, University Hospital of Psychiatry and University of Zurich, Zurich, Switzerland

**Keywords:** chronic stress, emotional memory, ethocybin, glutamate synapse, mouse, psilocybin

## Abstract

There is considerable interest in the concept that psychedelic compounds might facilitate reorganization of emotional processing with therapeutic benefit; in particular, amelioration of excessive focus on past, current and potential-future aversive events, a dominant emotional-cognitive state in stress-related affective disorders. Translational animal models can advance understanding of specific aspects of this concept, namely, those relating to subconscious emotional memories and their neurobiology. In male mice, chronic social stress (CSS) leads to excessive tone/context-foot shock Pavlovian aversion learning (PAL) and memory (PAM), measured as time spent in freezing behavior, and provides a model for generalized excessive focus on aversion. Sub-chronic administration of SSRI antidepressant between PAL and PAM reverses the excessive PAM of CSS mice. Here we applied the model to investigate acute and sustained behavioral and neurobiological effects of psilocybin (PSY) and ethocybin (ETH). In control male mice, both psychedelics at 3 mg/kg per os resulted in 1–2 h of blood-brain exposure to the respective major metabolites psilocin and ethocin, and to head twitch responses. In the main experiment, mice underwent either 15-day CSS or control handling (CON), followed 2 days later by PAL, 1 day later by PSY, ETH or vehicle (VEH), and 3 days later by PAM testing and then brain collection. Relative to VEH, PSY and ETH led to increased PAM expression as more time spent in freezing behavior during tone/context presentation. In a subset of these same mice, there was no psychedelic effect on densities of the two glutamatergic synaptic proteins investigated, vGluT1 or Homer1 in prelimbic cortex or amygdala. This study demonstrates that PSY and ETH lead to a sustained increase in aversive emotional memory expression in male mice. A similar effect in humans with stress- and trauma-related affective disorders might increase the psychotherapeutic accessibility of such memories.

## 1 Introduction

Monoamine reuptake inhibition has been the primary antidepressant mechanism of action (MoA) since the 1970s, with current first-line pharmacotherapies being selective reuptake inhibitors of serotonin (SSRI), norepinephrine (NRI) or serotonin and norepinephrine (SNRI) (Cipriani et al., 2018). However, a substantial proportion of patients prescribed these drugs fail to achieve remission, including those with major depressive disorder (MDD), an anxiety disorder (AD) or post-traumatic stress disorder (PTSD) (Rush et al., 2006; Rush et al., 2009; Perez Benitez et al., 2012; Kelmendi et al., 2017; Ansara, 2020). In the search for much-needed novel therapeutic MoAs, attention has turned to classic psychedelic compounds, building on the psychedelic-assisted psychotherapy research conducted in the 1950s-70’s (Leuner, 1994; Vollenweider and Preller, 2020; Weston et al., 2020). Examples of classic psychedelics, also known as serotonergic hallucinogens, are lysergic acid diethylamide (LSD), *N,N*-dimethyltryptamine (DMT), 4-phosphoryloxy-*N,N*-dimethyltryptamine (psilocybin, PSY; 4-PO-DMT) and its analogue 4-phosphoryloxy-*N,N*-diethyltryptamine (ethocybin, ETH; 4-PO-DET). These compounds are potent serotonin (5-HT) receptor agonists and exert many of their characteristic acute psychological effects through activation of the serotonin 2A receptor (5-HT_2A_R) (Vollenweider et al., 1998; Preller et al., 2017; Klein et al., 2020; Kwan et al., 2022). The 5-HT_2A_ receptor is expressed in both cortical and subcortical regions of mammalian brains (including rodent and primate), primarily on glutamatergic pyramidal neurons and to some extent on GABAergic interneurons (Jakab and Goldman-Rakic, 1998; Puig et al., 2003; Santana et al., 2004).

Of critical importance to discovering and validating novel psychotropic MoAs is a clear, mechanistically-grounded rationale that specifies the: (1) major symptom or process, rather than an entire heterogeneous disorder, being targeted; (2) neural circuitry that underlies this process; (3) putative pathophysiology within one or more of the brain regions contributing to this circuitry and symptom/process. The research domain criteria framework (RDoC; www.nimh.nih.gov/research) has provided substantial impetus to this psychological-neurobiological approach. Of the RDoC domains, negative valence systems–i.e., systems responsible for responses to aversive events/stimuli, actual or potential, acute or chronic–are of most relevance to conceptualizing a MoA rationale for psychedelic drugs (Nichols et al., 2017; Raut et al., 2022). In stress-related affective disorders, emotional-cognitive models emphasize that maladaptive aversion processing is predominant; this includes, for example, excessive responsiveness and Pavlovian conditioning to aversive stimuli; persistent rumination on negative or distressing thoughts or feelings; and perceived feelings of uncontrollability of aversive stimuli (Disner et al., 2011; Beckers et al., 2023). At the neural circuitry level, there is evidence for aversion stimulus-related increased activity in thalamus and amygdala and decreased activity in some prefrontal cortex subregions, consistent with impaired-top-down regulation of negative affect (Disner et al., 2011; Beckers et al., 2023). Psychedelics and/or their active metabolites, binding at 5-HT_2A_R on pyramidal glutamatergic neurons in prefrontal cortex, induce a characteristic psychedelic state marked by altered self-consciousness/awareness and changes in sensory, emotional and cognitive processing (Vollenweider and Preller, 2020). It has been hypothesized that this acute 5-HT_2A_-mediated experience and the sustained neurobiological consequences thereof, could constitute a window of elevated responsiveness to psychotherapeutic processing of aversive memories and feelings (Kwak et al., 1993; Vollenweider and Preller, 2020; Yaden et al., 2022; Grunder et al., 2024).

At the cellular level, psychedelic activation of 5-HT_2A_R on apical dendrites located on cortical glutamatergic pyramidal neurons increases their firing and promotes glutamate release onto ionotropic (e.g., AMPA, NMDA) and metabotropic (e.g., mGluR1) receptors of post-synaptic, often glutamatergic, neurons (Marek, 2018; Shao et al., 2025). The resultant acute increases in signaling pathway activity and synaptic-gene and -protein expression can lead to sustained increases in spinogenesis and synaptogenesis, that in turn support synaptic plasticity (Ly et al., 2018; Vollenweider and Preller, 2020; Aleksandrova and Phillips, 2021; de Vos et al., 2021; Shao et al., 2021; Sonda et al., 2025). Occurring primarily in prefrontal cortex, such changes might restore top-down control on sub-cortical regions including amygdala, commensurate with increased cognitive and decreased emotional processing of aversive stimuli and extinction of aversive memories (Senn et al., 2014; Vollenweider and Preller, 2020; Berboth and Morawetz, 2021). Somewhat contrary to this concept, in MDD patients a single dose of PSY resulted in increased fMRI amygdala reactivity to fearful *versus* neutral faces at 1-day post-treatment. The PSY administration was combined with psychotherapy, and greater amygdala responsiveness to fearful faces predicted higher remission and treatment response at 1-week post-PSY + psychotherapy (Roseman et al., 2018). This finding suggests that, in some contexts, the dominant effect of psychedelics is to increase the synaptic plasticity of bottom-up amygdala inputs to cortical regions, thereby facilitating emotional-cognitive access to aversive memories and their subsequent extinction.

Experiments that can disentangle these various neurobiological-psychological hypotheses of the MoA(s) of psychedelic drugs are essential, and include cause-effect studies in valid animal models. The absence of self-consciousness/-awareness in laboratory animal species means that they can contribute evidence on subconscious states specifically (LeDoux and Pine, 2016). This species difference is exemplified by the respective acute hallucinogenic effects of 5-HT_2A_R agonism: the head twitch responses (HTRs) of rodents (Halberstadt et al., 2020) contrast with the changes in self-, emotional and cognitive processing in humans (Vollenweider and Preller, 2020). Despite this major difference, valid rodent-human translational paradigms for studying adaptive and stress-induced excessive subconscious and conscious aversion processing exist and can be applied in psychedelic research. Major among these is Pavlovian aversion (“fear”) learning and memory (LeDoux and Pine, 2016). In rodents, typically tone and foot-shock are deployed as conditioned stimulus (CS) and unconditioned stimulus (US), respectively, and freezing behavior constitutes the conditioned response. To date, several mouse studies have assessed effects of PSY and analogues on Pavlovian aversion memory expression and extinction. Most have administered the compound shortly (∼30 min) before CS memory expression/extinction testing: to summarize, 1.0–2.5 mg/kg reduces time spent freezing, although the HTRs on-going at this time point confound the reliability of this readout (Catlow et al., 2013; Hagsäter et al., 2021; Du et al., 2023; Woodburn et al., 2024; Rogers et al., 2025). With respect to longer delays between administration and memory testing: PSY at 2 mg/kg reduced freezing in a memory test directly post-administration, but was without effect (whether given directly before or after this first test) in tests at 24–48 h post-administration (Lu et al., 2025). Psilocybin reduced freezing in repeated tests at 30 min, 1 day and 7 days post-administration, with the long-term effects specific to 1 mg/kg and absent at 0.5 or 2 mg/kg (Woodburn et al., 2024), and in repeated tests at 30 min, 1 day and 6 days post-administration at 2.5 mg/kg and not at lower doses (Du et al., 2023). Only one study has investigated PSY effects on Pavlovian aversion memory in mice having undergone chronic stress: 3 mg/kg reduced freezing in a memory test at 30 min post-administration, but again the overlap with HTRs necessitates caution (Razidlo et al., 2025).

We have developed a mouse model of chronic social stress (CSS)-induced excessive aversion processing, measured as increased time spent freezing during both tone/context-foot shock Pavlovian aversion learning (PAL) and subsequent Pavlovian aversion memory (PAM) expression/extinction to tone-CS + context, compared with control (CON) mice (Sigrist et al., 2024; Poggi et al., 2025). Sub-chronic administration of SSRI between PAL and PAM leads to normalization of excessive PAM expression in CSS mice, demonstrating predictive validity (Poggi et al., 2025). In addition, the model is sensitive to novel molecular targets, including somatostatin receptor agonism (Adamcyzk et al., 2022) and transient receptor potential canonical channel inhibition (Just et al., 2018; Poggi et al., 2025; Poggi et al., 2026), suggesting involvement of amygdala (AMYG) and medial prefrontal cortex (mPFC) in the neural circuitry mediating the CSS-PALM effect. In the current mouse study, we assessed a single oral dose (3 mg/kg) of either PSY or ETH in terms of the acute pharmacokinetic profile of their major metabolites in blood and brain and the acute pharmacodynamic change in HTRs. In the main experiment, we investigated whether this dose of PSY or ETH exerts sustained effects on PAM in CON and CSS mice, and whether it leads to changes in pre-synaptic vesicular glutamate transporter 1 and post-synaptic Homer 1 in mPFC and AMYG. Both PSY and ETH led to increased PAM expression at 3 days post-administration, in the absence of any change in the two synaptic proteins in mPFC or AMYG. This demonstration of a sustained activating effect of psychedelics on PAM expression in a mouse model adds substantially to current evidence for the potential neurobehavioral relevance and MoA of classic psychedelics in the treatment of excessive aversion processing in stress-related affective disorders.

## 2 Materials and methods

### 2.1 Animals

Experiments were conducted with male C57BL/6JRj mice aged 10–12 weeks at experiment onset. Mice were bred in-house, with breeding pairs obtained from Janvier Laboratories (Le Genest-Saint-Isle, France). Each breeding pair of mice contributed 2 littermates to the study. After weaning, mice were maintained in littermate pairs, unless single housing was necessary for the purpose of the experiment (see below for details). Cages (33 × 21 × 14 cm, containing sawdust, sleeping house, tissue bedding) were ventilated individually, and food (Complete Pellet, Kliba Nafag, Granovit AG, Switzerland) and water were available continuously. Mice were maintained under a 12 h: 12 h inverted day-night cycle with lights off at 07.00–19.00 h. Temperature was kept at 21 °C–23 °C and humidity at 50%–60%. Experiments were conducted between 09:00–16:00 h. In each experiment, mice were allocated to treatment groups in their littermate pairs using block randomization in batches of 2 or 3 to ensure equal group sizes, using an automated randomization generator. The first phase of each experiment involved handling each mouse for 5 min per day on three consecutive days to adjust them to human contact. The study was conducted under an experimental permit (ZH-182/2020) issued by the Veterinary Office, Canton Zurich, Switzerland. The sample size per treatment group in each experiment was based on our extensive experience with conducting pharmacological experiments with the chronic social stress paradigm and Pavlovian aversion learning-memory and/or neuroanatomical measures: n = 12 and n = 8, respectively, yield moderate to large effect sizes and identify stressor × compound interaction effects [e.g., (Azzinnari et al., 2014; Fuertig et al., 2016; Cathomas et al., 2019; Adamcyzk et al., 2022; Poggi et al., 2025)].

### 2.2 Compounds

Psilocybin and ethocybin (Carbogen Amcis AG, Bubendorf, Switzerland) were obtained and used for animal research purposes under permits issued by the Federal Office for Health, Bern, Switzerland, and the Federal Department for the Interior, Bern, Switzerland, respectively. The vehicle (VEH) was 0.5% hydroxyethyl cellulose (Sigma-Aldrich, #09368) in ddH_2_O. Each compound was prepared on each day of use and administered to mice using an oral gavage in a volume of 5 mL/kg body weight. The study dose of 3 mg/kg corresponds allometrically to a human-equivalent dose of 0.24 mg/kg (Nair and Jacob, 2016), i.e., a moderate-to-high clinical dose of approximately 15–20 mg for a 60–80 kg adult (von Rotz et al., 2023). A dose equivalent to a moderate-to-high human dose was selected given that we were investigating for sustained effects of a single dose of psychedelic.

### 2.3 Experiment 1. acute psychedelic and major metabolite pharmacokinetics in blood and brain

#### 2.3.1 Blood and brain sampling

Mice in littermate pairs were assigned to PSY (n = 10 mice), ETH (n = 10 mice) or VEH (n = 2 mice). Mice were placed singly in a restraint tube that allowed access to the tail. An initial baseline (time = 0 min) blood sample was collected by making a small incision in the lateral tail vein and collecting about 60 µL blood into an EDTA-coated capillary tube (Microvette CB 300 EDTA K2E, Sarstedt) which was then placed on ice (Pryce et al., 2011). Compound or VEH was then administered (time = 1 min) and the mouse returned to its home cage. Further blood samples (each 60 µL) were collected at 15, 30 and 60 min in the case of n = 5 for PSY and ETH groups and at time points 15, 30, 60, and 120 min in the case of n = 5 for PSY and ETH and n = 2 VEH mice. Each sample was drawn from the same incision site, with mice maintained in the home cage between samplings. Directly after the final blood sample (60 min or 120 min), mice were deeply anaesthetized with pentobarbital (150 mg/kg i. p.) and perfused transcardially with PBS for 1 min to rinse out blood, followed by removal of the brain and placement on dry ice. Blood samples were centrifuged at 4 °C for 15 min at 780 × *g*, and the plasma (25–35 µL) pipetted off and stored at −80 °C. Brains were thawed and dissected into cortical and subcortical compartments; in a 1.5 mL tube (Eppendorf Protein LoBind), 100–200 mg brain tissue was homogenized with 2× (µL:mg) double distilled water using a disposable pestle (VWR); 100 µL homogenate was pipetted off and re-frozen. Thus, each mg of tissue corresponded to a final homogenate volume of approximately 3 µL (1 µL tissue + 2 µL water, assuming a tissue density of ∼1 g/mL). Consequently, a homogenate concentration of 1 ng/mL corresponds to 3 ng/g tissue. Concentrations measured in homogenate (ng/mL) were converted to tissue concentrations (ng/g) by multiplying by a factor of 3.

#### 2.3.2 Sample preparation and LC-MS/MS

Mouse plasma and homogenized brain samples were stored at −80 °C until analysis. Sample preparation and analysis were conducted based on previously published protocols for psilocin in human plasma (Kolaczynska et al., 2021; Thomann et al., 2024). This included the experimenter not being blinded with respect to sample identity, given that the procedure was fully automated. In brief, per plasma sample, 10 µL were precipitated with 200 µL methanol containing the internal standards psilocin-d_10_ (20 ng/mL) and tryptophan-d_5_ (100 ng/mL); per homogenized brain sample, 20 µL were extracted by adding 30 µL water and 150 µL internal standard solution. Following protein precipitation, all samples were centrifuged at 3,220 *g* for 30 min at 15 °C (5810 R centrifuge, Eppendorf), yielding protein-free supernatants. A modular liquid chromatography–tandem mass spectrometry (LC–MS/MS) system was used for analysis of PSY, ETH and their respective metabolites. Chromatographic separation was performed using a Symmetry C18 analytical column (3.5 μM, 100 Å, 4.6 × 75 mm; Waters, MA, United States) on a high-performance liquid chromatography (HPLC) system (Shimadzu, Kyoto, Japan), coupled to an API 5000 triple quadrupole tandem mass spectrometer (AB Sciex, Ontario, Canada). Mobile phases consisted of water and methanol, both supplemented with 0.1% formic acid. Total sample run time was 4.5 min. The two parent compounds, PSY and ETH, were monitored qualitatively using the transitions *m/z* 285.1 → 58.2 and *m/z* 313.1 → 86.2, respectively. As no signals corresponding to these parent compounds were detected in any study sample, these analytes were not included in the quantitative assay. Detailed mass spectrometric parameters and calibration ranges for psilocin and ethocin are given in Supplementary Table S1. Instrument control was carried out using Analyst (AB Sciex, version 1.7.2), and quantitative data analysis was performed with MultiQuant (AB Sciex, version 3.0.3).

### 2.4 Experiment 2. acute psychedelic effect on head twitch responses

At 2 days prior to the onset of testing, mice (n = 6 per compound or VEH) were caged singly and were adjusted to the cage set up used for testing: rather than the usual cage grid and lid, a sheet of transparent PVC was placed on top of the cage base for 60 min to allow for video recording from above. A counterbalanced cross-over design was used in which each mouse received both VEH and one of PSY or ETH (VEH-PSY or PSY-VEH; VEH-ETH or ETH-VEH), with a washout interval of 3–4 days. Directly after administration, the transparent PVC lid was placed on the cage base situated directly beneath a video camera. For 60 min post-administration, an experimenter blinded to compound/VEH scored head twitch responses (HTRs) continuously, i.e., each discrete and brief HTR was given a score of 1. The sessions were also video recorded, and a random sample of 10 sessions were re-scored for validation of reliability, by the same experimenter who was blinded to mouse identity and compound/VEH: intra-observer test-retest reliability expressed as a Pearson correlation was r = +0.985 (n = 10, 8 d.f.). For analysis, the 60 min were divided into 4 × 15 min periods.

### 2.5 Experiment 3. sustained psychedelic effects on Pavlovian aversion memory and specific glutamate synapse proteins in mPFC and AMYG

#### 2.5.1 Experimental design

Mice littermate pairs were assigned randomly and equably to stressor group (CON, CSS) such that n = 40 mice per group. Mice underwent CON or CSS on days 1–15, and this was followed by PAL testing on day 17. The per mouse mean tone CS/context-freezing score for the final 2 PAL CS-US trials was used to counterbalance littermate pairs equably to compound (VEH, PSY, ETH), such that n = 12–14 mice per study arm. On day 18, at 24 h post-PAL testing, compound was administered. On day 21, at 72 h after compound administration, PAM expression testing was conducted. At 90 min after the onset of the PAM test, mice were perfused-fixed for brain collection. The experiment was divided into three runs, with 4–6 mice per study arm per run.

#### 2.5.2 Chronic social stress

The CSS procedure is based on the resident-intruder paradigm (Azzinnari et al., 2014; Sigrist et al., 2024). Resident mice were singly caged, aggressive, ex-breeder CD-1 males weighing 40–60 g, obtained from Janvier Laboratories, with a separate cohort for each experimental run. A transparent, perforated divider was placed along the length of the home cage of each CD-1 mouse. On each of 15 days (experimental days 1–15), a BL/6 CSS mouse was placed in the same compartment as an unfamiliar CD-1 mouse and observed by the experimenter. Physical attack was timed up to a cumulative total of 30–60 s or mice remained together for 10 min maximum, whichever occurred sooner. Thereafter, the two mice were separated by placement on either side of the divider, and remained in distal (visual, olfactory, auditory) contact for 24 h. CSS mice were rotated each day between CD-1 mice cages, and were therefore exposed to 12–14 different such mice. The CSS procedure (Azzinnari et al., 2014; Sigrist et al., 2024) incorporates several animal welfare refinements relative to the widely-used protocol of chronic social defeat [CSD (Golden et al., 2011)]. Briefly, the daily cumulative duration of attack time is set at 60 s maximum, as timed by the experimenter, rather than the mice being placed together, unobserved, for 10 min. This, combined with the trimming of the lower incisor teeth of the CD-1 mice, markedly reduces the likelihood of bite wounds. This is important from animal welfare and scientific perspectives: the immune activation caused by bite wounds can impact on brain status and function, thereby compromising the relevance of the mouse model to human psychosocial stress and its effects on emotional stimulus processing. Trimming of the continuously growing lower incisors is conducted every third day and involves brief anesthesia with 4% isoflurane followed by removal of the pointed tip of the lower incisor teeth using a rodent tooth cutting forceps. Food pellets are cut into smaller pieces to ensure that the CD-1 mouse is able to eat them. The CSS and CON mice are weighed each day and if body weight would be below 90% of pre-CSS body weight on any 2 days the mouse would be removed from the experiment. If a CSS mouse would endure a surface (skin only) bite wound on any 3 days it would be removed from the experiment. If a CSS mouse would display inactivity in response to handling on any day it would be removed from the experiment. The CD-1 mice are weighed each day and if body weight would be below 90% of pre-CSS body weight on any 2 days the mouse would be removed from the experiment. If a CD-1 mouse would display stereotypic behavior on any 1 day it would be removed from the experiment. In this study, using these humane endpoints, no BL/6 CSS mouse or CD-1 mouse had to be removed from the experiment. After the 15-day procedure, each BL/6 mouse remained in the cage of the same CD-1 mouse, separated in adjacent compartments and without any further attacks, until the termination of the experiment. Control (CON) mice were kept as pairs of littermates and handled for weighing on each experimental day.

#### 2.5.3 Pavlovian aversion learning and memory

Pavlovian aversion learning-memory (PALM) testing (Poggi et al., 2025) was conducted using a 4-unit MultiConditioning System (TSE Systems), where each unit comprised: an attenuation chamber equipped with low-level white light (10 lux) and low-level white noise; a test arena (context) with lid; a floor comprising stainless steel rods for delivery of electrified foot-shocks that serve as the unconditioned stimulus (US); a loudspeaker for delivery of tone that serves as the conditioned stimulus (CS); an infra-red beam system for detection of activity or inactivity including, respectively, the unconditioned responses (UR) of running and jumping to the US and the conditioned response (CR) of freezing to the CS and/or context. On day 17, mice were placed in the context for 6 min habituation without CS or US. Directly thereafter PAL testing was conducted, comprising 6 tone-foot shock pairings (CS/context-US trials), using 20 s tone at 5 kHz and 85 dB, and 2 s foot shock at 0.15 mA concurrent with the final 2 s of tone. Inter-trial intervals (ITIs) between successive CS/context-US trials were 120 s. Aversion learning was measured as the percentage of the 18 s CS and the 120 s ITI the mouse spent in the CR state of freezing. The rate and asymptote of freezing acquisition are directly and primarily dependent on the perceived aversive salience of the US (Rescorla, 1988). Mice were returned to the CD-1 cage (CSS) or home cage (CON) directly after PAL testing. On day 21, PAM expression/extinction testing was conducted, and comprised placement in the same context as for PAL testing, adjustment to the context for 2 min (due to the software parameter settings, freezing data could not be collected during this period), followed by presentation of ten 30 s CS/context trials interspersed with ITIs of 90 s. Expression of aversive CS/context and context memory were measured, respectively, as percentage of the 30 s CS and 90 s ITI spent in the state of freezing. Thus, 10 CS/context trials and the nine ITIs were the prespecified periods for assessment of PAM expression/extinction, as per usual for the CSS-PALM model [e.g., (Azzinnari et al., 2014; Fuertig et al., 2016; Just et al., 2018; Adamcyzk et al., 2022; Sigrist et al., 2024; Poggi et al., 2025; Poggi et al., 2026)]. Mice were returned to the CD-1 cage (CSS) or home cage (CON) directly after PAM testing and anaesthetized and perfused-fixed at 90 min after PAM testing onset. As the collection of these behavioral data was completely automated, the experimenter was not blinded with respect to mouse allocation to stressor and compound groups.

For the *ex vivo* analysis of the effects of stressor and compound on glutamate synapse proteins, the prespecified design was to conduct the experiment with 8 of the 12 mice per group, and these mice were selected by ranking them highest to lowest in their groups according to mean % time spent freezing during PAM CS/context trials, and the mice ranked 1–8 per group were included in the *ex vivo* experiment.

#### 2.5.4 Immunofluorescence staining of glutamate synaptic proteins vGluT1 and Homer 1

##### 2.5.4.1 Brain perfusion-fixation and histology

Mice were deeply anaesthetized by injection of pentobarbital (150 mg/kg i.p.) followed by transcardial perfusion-fixation, firstly with phosphate-buffered saline (PBS, pH 7.4, 20 mL in 1 min) and then freshly prepared, ice-cold paraformaldehyde (PFA, 4% in 0.4M sodium phosphate buffer, 60 mL in 3 min). Brains were extracted and placed in 4% PFA for post-fixation for 2 h. They were then rinsed with PBS and underwent a cryoprotective sucrose cascade of placement in 10%, 20% and 30% sucrose/ddH_2_O solution, each for 24 h at 4 °C. Brains were then embedded in Tissue-Tek® O.C.T.^TM^ in a Cryomold® (25 × 20 × 5 mm), frozen on powdered dry ice and stored at −80 °C until sectioning. Using a microtome (Leica) at around −30 °C, brains were sectioned coronally, anterior to posterior, at 40 µm. Sections collected at bregma 1.80–2.00 mm included mPFC (region of interest (ROI): prelimbic cortex, PrL), and at bregma −1.80 to −2.00 mm included AYMG (ROIs: lateral amygdala, LA; basal amygdala, BA); a mouse brain atlas was used in identifying these bregma levels (Paxinos and Franklin, 2001). Sections were first collected in PBS and then transferred into the wells of a 96-well plate containing 200 µL tissue collection solution [TCS; glycerine and ethylene glycol in 0.2 M phosphate buffer (Sigma-Aldrich)] and stored at −20 °C until immunofluorescence staining.

##### 2.5.4.2 Immunofluorescence staining

Sections for each brain region were processed separately. The experimenter was blinded with respect to the individual mouse identity, and therefore stressor and compound group allocation, of the brain sections. For quantification of pre- and post-synaptic proteins, and of their colocalization at what were inferred to be synaptic puncta, immunofluorescence co-staining was conducted. Vesicular glutamate transporter 1 (vGluT1) was of interest as a pre-synaptic protein, and post-synaptic density scaffolding protein (Homer1) as a post-synaptic protein. Per mouse, two coronal sections for each of PrL and AMYG were selected for immunofluorescence staining. The 96 sections per brain region (2 stressors x 3 compounds x 2 sections x n = 8 per group) underwent immunofluorescence in three counterbalanced runs. In each run, a negative control section with PrL was included.

Pilot immunostaining demonstrated that optimal signal coverage for vGluT1 and Homer1 were obtained by incubating sections with the former in the absence of the latter for 24 h, followed by a period of incubation with both antibodies for a further 24 h (Wicki et al., 2026). The immunofluorescence staining protocol spanned three consecutive days (days 1–3). The following staining steps were conducted using 24-well tissue culture plates (Falcon®), with each well containing the 2 sections from one brain region and mouse. For each washing step and incubation period, the 24-well plates were placed on a shaker (120 rpm) at room temperature (RT). On day 1, to remove all tissue collection solution, sections were washed in 1× PBS (pH 7.4) for 1 h (3 × 20 min). Sections were then transferred to blocking solution of 20% normal goat serum (NGS) with 1× PBS (300 µL/well) for 1 h. They were then transferred to a solution of guinea pig polyclonal anti-vGluT1 (240 µL/well, 1:500, #135304, Synaptic Systems) in 0.3% Triton in 1× PBS and 10% NGS, and incubated for 24 h at 4 °C. On day 2, a solution containing 0.3% Triton in 1× PBS, 10% NGS and rabbit polyclonal anti-Homer1 (10 µL/well, 1:20, #160002, Synaptic Systems) was added to the wells, giving a final anti-Homer1 concentration of 1:500, and the sections were incubated for 24 h at 4 °C. On day 3, unbound primary antibodies were removed by transferring the sections to 1× PBS and washing for 1 h (3 × 20 min). Sections were then returned to the well plates and incubated for 2 h with 300 µL/well of a solution containing the fluorophore-conjugated secondary antibodies, goat anti-guinea pig DyLight 650 (1:250, #SA5-10097, Invitrogen, Thermo Fisher Scientific) and goat anti-rabbit Alexa Fluor 488 (1:250, #A11008, Invitrogen, Thermo Fisher Scientific), in 0.3% Triton in 1× PBS and 10% NGS. This fluorophore incubation and all subsequent steps were performed in the dark. To remove unbound secondary antibodies, the sections were transferred to 1× PBS and washed for 1 h (3 × 20 min). The sections were mounted on microscope slides (Superfrost™ Plus Adhesion Microscope Slides, New Erie Scientific LLC, Epredia) with a solution containing 50% 1× PBS and 50% dH_2_O, air dried, dipped in dH_2_O, and air dried again. For cell nucleus staining, DAPI Fluoroshield Mounting Medium (Fluoroshield™ with DAPI, Sigma-Aldrich) was used. Cover slips (24 × 50 mm, New Erie Scientific LLC, Epredia) were mounted on the immunostained sections, and slides were stored in a closed microscopy box maintained at 4 °C until image acquisition within 1–5 days.

##### 2.5.4.3 Image acquisition

Image acquisition was conducted 1–5 days after completion of immunofluorescence staining (Wicki et al., 2026). The regions of interest (ROIs) were layers II/III of the prelimbic cortex (PrL) of the mPFC and the lateral nucleus (LA) and basal nucleus (BA) of the AMYG. With the aid of a mouse brain atlas (Paxinos and Franklin, 2001) and at ×10 magnification using the DAPI channel, the ROIs were identified: for PrL, its dorsal boundary and the lateral distance from the midline provided important landmarks; for LA, an intersection at one-third of the dorsal-ventral length of AMYG and the LA’s medial-lateral midline provided the ROI center, and for the BA, an intersection at the ventral limits of the white-matter capsules bordering AMYG and the BA’s medial-lateral midline provided the ROI center. The PrL, LA and BA ROI images were acquired for each hemisphere (Supplementary Figures S2 and S3). Imaging was conducted using an automated inverse confocal laser scanning microscope (Leica SP8) running LSX software. The Leica SP8 objective used was HCX PL APO 37 °C CS2 63×/1.3 NA glycerol. With the hybrid (HyD) detectors in photon counting mode, the fluorescence channel DyLight 649 was used for vGluT1 with the HyD detector set at the range 646–800 nm and laser power at 10%, and the fluorescence channel ALEXA 488 was used for Homer1 with the HyD detector set at 500–562 nm and laser power at 8%. Due to uneven distribution (“sandwich effect”) of the vGluT1 signal, the well-impregnated surface (*z*-size = 8.70 µm) of the brain section that was in contact with the microscope slide was imaged specifically, with a *z*-step size of 0.30 µm and 30 acquisition steps. By choosing 1,024 × 1,024 as format, scan speed of 400 Hz with a bidirectional scan direction X, zoom of 3, frame accumulation of 2 and a pinhole of 1 AU, ROI acquisition was at a final image size of 61.51 × 61.51 µm and pixel size of 60.13 × 60.13 nm. The *z*-step size of 0.30 µm was 2× that proposed by the Nyquist calculator such that we slightly under-sampled but were still within the valid range according to Nyquist sampling.

##### 2.5.4.4 Synaptic puncta detection and colocalization analysis

vGluT1 and Homer 1 synaptic puncta detection and 3D colocalization (vGluT1-Homer1) analysis were performed on fluorescence image *z*-stacks using custom MATLAB routines ((Wicki et al., 2026), available on request). Images from the two fluorescence channels were processed independently to quantify both protein-specific puncta parameters and colocalization metrics.

For image preprocessing and puncta detection, image stacks were first normalized to peak intensity ( $I/\max\left(I\right)$ ) to correct for variations in fluorescence intensity across samples. Background fluorescence was selectively removed using a 2D top-hat filter (*imtophat*, disk element radius r=5 pixels ) to enhance the contrast of small, bright puncta. To suppress noise while preserving structural edges, 2D slice-by-slice Wiener adaptive filtering (*wiener2*, 3 × 3 spatial window, 1 iteration) was applied. Channel-specific puncta sizes were estimated iteratively across a candidate diameter range of 5–10 pixels. Puncta were detected on Maximum Intensity Projections (MIPs) using Laplacian of Gaussian (LoG) blob detection. Projections were contrast-adjusted (*imadjust*), complement-inverted (DarkBackground = true), and median-filtered (*medfilt2*). Candidate blobs were identified as local spatial maxima relative to a 4 × 4 pixel square dilation neighborhood, excluding border regions within ⌈diameter/2⌉ pixels and filtering out signals with rescaled intensities ≤0.2 (QualityFilter = 0.2).

Puncta colocalization was determined by evaluating the 3D volumetric overlap of puncta between the two channels. Candidate puncta pairs were classified as colocalized if they satisfied three quantitative criteria:

1. Centroid Distance: A maximum 2D XY Euclidean distance ≤5 pixels .
2. Axial Overlap: A positive axial overlap ( coincideLen>0 ), determined by fitting 1D Gaussian distributions ( 3×3 spatial window) to establish Z -axis signal boundaries ( $t_{\text{lead}},t_{\text{trail}}$ ).
3. Volumetric Cutoff: Physical volumetric overlap exceeding a fixed minimum volume threshold: ${\text{Threshold}}_{\text{vol}}=4\cdot \text{zPixelDim}\times 4\cdot \text{xPixelDim}\times 4\cdot \text{xPixelDim }\mu m^{3}$ .

where 2D spatial masks derived from rotated 2D Gaussian fitting were aligned *via* coordinate circular shifting (*circshift*) and combined *via* logical intersection. Inter-puncta spatial relationships were further characterized using nearest-neighbor Euclidean distances and global Jaccard similarity indices computed on centroid-dilated binary masks.

To ensure that Z -axis sampling density did not introduce quantification artifacts or miss small synaptic puncta, the analysis pipeline utilized sub-voxel continuous modeling rather than discrete voxel thresholding. Standard voxel-counting approaches are vulnerable to Z -spacing variations because an optical slice missing the center of a small punctum can cause sharp, stepwise drops in detected volume or binary overlap. To prevent this, continuous 1D Gaussian profile fitting was incorporated along the Z -axis. For every candidate punctum, the algorithm sampled a 3 × 3 pixel spatial window around the centroid across all Z -slices and fitted a continuous 1D Gaussian distribution. From this continuous fit, the leading ( $t_{\text{lead}}$ ) and trailing ( $t_{\text{trail}}$ ) boundaries of the fluorescence intensity profile were derived in real physical space ( μm ). The axial overlap length ( coincideLen ) between paired channels was then calculated as:

$$\text{coincideLen}=\left(\min\left(t_{\text{trail},1},t_{\text{trail},2}\right)-\max\left(t_{\text{lead},1},t_{\text{lead},2}\right)\right)\times \text{zPixelDim}$$

Because signal boundaries ( $t_{\text{lead}},t_{\text{trail}}$ ) were estimated continuously from the fitted Gaussian curve rather than rounded to discrete slice indices, the estimated overlapping volume remained smooth and mathematically stable. Even if a punctum fell between physical Z -planes or spanned few optical slices, the underlying Gaussian model interpolated the axial intensity distribution, inherently cushioning the pipeline against Z -undersampling artifacts and preventing discrete slice-clipping errors.

Image processing, blob detection, and colocalization routines were executed automatically without prior information regarding experimental conditions. Primary quantitative outputs included puncta counts, colocalization volumes, and fluorescence intensities. For statistical analysis, the mean value across four sampled sections (2 hemispheres × 2 sections per hemisphere) was calculated for each mouse and ROI.

### 2.6 Statistical analysis

Each breeding pair of mice contributed 2 littermates to the study and the 2 littermates were treated as independent subjects and as the units of analysis, with data visualization indicating that per group differences between littermates and non-littermates were similar. In experiment 1, the pharmacokinetic data presented are descriptive and exploratory. Formal pharmacokinetic parameters were not calculated, and no inferential statistical comparisons between PSY and ETH or between brain regions were performed. Values below the analytical quantification range were treated as zero in the graphs and summary calculations. In experiment 2, the head twitch response data were analyzed using Prism (GraphPad Prism 9). The ROUT test reported an absence of outlier values and values were normally distributed according to the D’Agostino-Pearson test. Compound effects on HTRs were analyzed using paired and unpaired *t* tests: the 3–4-day washout period was more than adequate to ensure, in mice that received PSY or ETH first and VEH second, that there was no compound carryover effect, so it was not necessary to model order of compound-vehicle administration. In experiment 3, PALM behavioral freezing data were analyzed with SPSS (IBM SPSS Statistics, version 31) and neuroanatomical data with Prism. With respect to PAL and PAM freezing data, boxplot analysis did not identify any outlier values, and values were normally distributed according to the Shapiro-Wilk test. For each of PAL and PAM, and within each of these for CS trials and inter-trial intervals (ITIs), data were analyzed using general linear models (GLMs), with analysis of variance (ANOVA) models including between-subject factors of stressor (CON, CSS) and compound (VEH, PSY, ETH) and a within-subject factor (repeated measure) of trials. As a first step, ANOVA models included experimental run (1, 2, 3) as an additional between-subject factor; there was no significant effect involving run and it was excluded from the main analysis to maintain statistical power. Mauchly’s test of sphericity was non-significant in the case of each ANOVA. In the case of statistical significance requiring *post hoc* analysis, Sidak’s multiple comparisons test was used. Effect size was measured as eta squared (η^2^), with 0.01, 0.06 and 0.14 taken as the thresholds for small, medium and large effect sizes, respectively. In addition, for PAM freezing, given the identity of some small differences between compound-allocation groups based on PAL data, analysis of covariance (ANCOVA) was conducted, to take individual differences in PAL freezing into account when assessing compound effects on PAM freezing: In the case of PAM CS data, either PAL mean freezing in CS trials 5–6 or in ITIs 4–5 was included as a covariate; in the case of PAM ITI data, PAL mean freezing in ITIs 4–5 was included as a covariate. Finally, although the experiment was not designed to do so with respect to statistical power, a *post hoc* analysis was conducted in which litter, rather than mouse, was used as the unit of analysis: a linear mixed model was applied with CS trials, Stressor and Compound as fixed effects and litter as a random effect. For synaptic marker immunofluorescence data, obtained with the 8 of the 12 mice per group with the highest PAM mean freezing scores for CS trials 1–10, ANOVAs with between-subject factors of stressor and compound were conducted in Prism. As a first step, ANOVA models included staining run (1, 2, 3) as an additional between-subject factor; there was no significant effect involving run and it was excluded from the main analysis. Homoscedasticity plots of residuals were checked visually and with Spearman’s rank correlation coefficient test. All statistical tests were two-tailed, and statistical significance was set at p ≤ 0.05, even in the case of multiple outcomes being tested, in line with the exploratory nature of the study. All data were graphed using Prism; data are reported as mean ± standard error of the mean (SEM) or as individual data points and mean. Illustrations were created with BioRender.com.

## 3 Results

### 3.1 Experiment 1. psychedelic acute pharmacokinetics in blood and brain

The aim of this experiment was to establish and compare the blood and brain exposure profiles of PSY and ETH and their psychoactive metabolites psilocin and ethocin across 2 h post-administration, whereby the data are descriptive and exploratory. In blood plasma and cortical and sub-cortical brain tissue, PSY and ETH were undetectable, even in the blood sample collected at the 15-min sampling time point. Psilocin, as the major metabolite of PSY, and ethocin, as the major metabolite of ETH, displayed similar absolute concentrations to each other, in both plasma and brain tissues (Figures 1A–D). In plasma, the peak exposure of psilocin and ethocin was observed at time points 15–30 min and then declined, with levels relatively low at 120 min. In brain tissue, exposures were similar in cortex and sub-cortex, and whilst mean levels were 600–900 ng/g tissue at 60 min they were relatively low at 120 min. No psilocin or ethocin was detected in VEH-injected mice (c < LLOQ).

**FIGURE 1:**
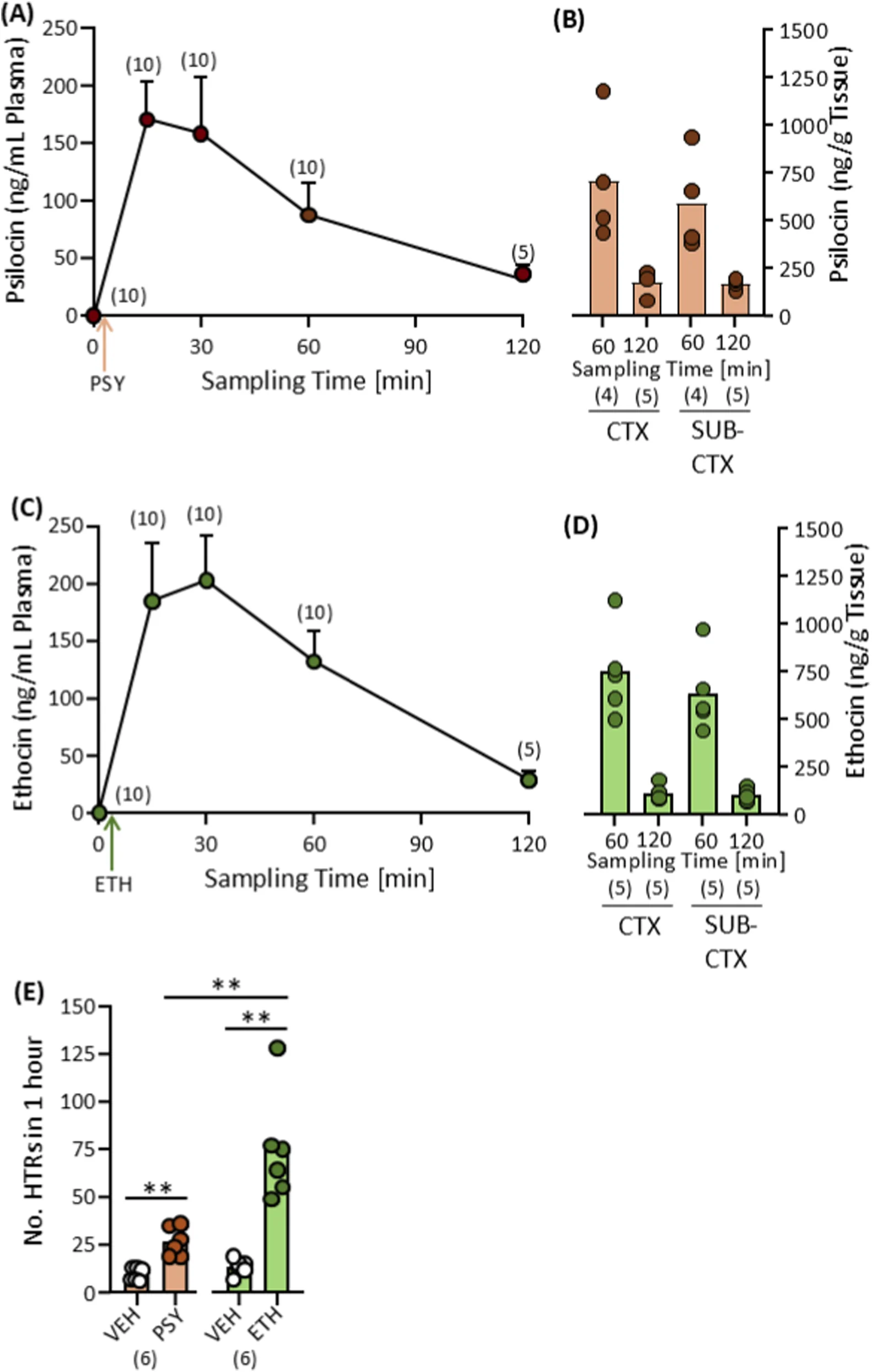
Psychedelic pharmacokinetics in terms of blood and brain exposure profiles of their major psychoactive metabolites and pharmacodynamics in terms of head twitch responses. Mice (n = 10 per compound) were administered PSY or ETH at 3 mg/kg per os at min 1. Tail vein blood samples were collected rapidly at either min 0, 15, 30, 60 (n = 5, 5) or min 0, 15, 30, 60 and 120 (n = 5, 5). Directly after the final blood sampling, mice were deeply anaesthetized and the brain removed and separated into cortical and subcortical compartments. Blood plasma and brain tissue samples underwent LC/MS-MS. **(A)** Psilocin levels in blood plasma at min 15–120 post-PSY administration. **(B)** Psilocin levels in brain tissue, cortex (CTX) and subcortex (SUB-CTX), at min 60 or 120 post-PSY administration. Collection of the brain of 1 mouse was overlooked at timepoint 60 min. **(C)** Ethocin levels in blood plasma at min 15–120 post-ETH administration. (D) Ethocin levels in brain tissue–cortex (CTX) and subcortex (SUB-CTX) – at min 60 or 120 post-ETH administration. **(E)** Mice (n = 6 per experiment), using a cross-over, counter-balanced design with a 3–4-day washout period, were administered per os either PSY at 3 mg/kg and VEH or ETH at 3 mg/kg and VEH, and observed throughout the subsequent 1 h for continuous scoring of head twitch responses. PSY induced an increase in the acute frequency of HTRs (paired t-test: t = 6.77, df = 5, p = 0.001), as did ETH (paired t-test: *t* = 5.93, df = 5, p = 0.002). Furthermore, ETH induced a higher HTR frequency than did PSY (unpaired t-test: *t* = 4.00, df = 10, p = 0.003).

### 3.2 Experiment 2. psychedelic acute pharmacodynamics as head twitch responses

The aim of this experiment was to investigate whether a single oral dose of PSY or ETH at 3 mg/kg would elicit an increase in the number of HTRs during 1-h post-administration. With each compound, mice displayed an increase in the frequency of HTRs relative to VEH (Figure 1E). Furthermore, mice that received ETH emitted more HTRs than mice that received PSY. When HTR frequency was assessed in four 15-min blocks, there was a small monotonic decrease across blocks in the case of PSY (min 1–15: 7.3 ± 2.6 (mean ± SD); min 46–60: 5.2 ± 0.7) and ETH (min 1–15: 22.5 ± 11.4; min 46–60: 14.5 ± 6.1) and not VEH (min 1–15: 2.1 ± 1.3; min 46–60: 2.8 ± 1.8).

### 3.3 Experiment 3. psychedelic sustained effects on Pavlovian aversion memory in the absence of effect on specific cortico-limbic glutamate synapse protein levels

In this main experiment, mice underwent CSS or CON handling on days 1–15 after which CSS mice remained in the cage of the same CD-1 stressor mouse without further attack until the end of the experiment (Figure 2A). The overall daily mean duration of attack per CSS mouse was 46.4 ± 2.8 s and attack durations were similar across the three runs of the experiment. In the CSS-PALM model, the stages PAL and PAM are conducted in the same context (arena), such that PAM freezing during CS-tone presentations represents a combination of the memories of CS- and context-foot shock conditioning (Sigrist et al., 2024). On day 17, all mice underwent PAL. Directly prior to PAL, mice underwent habituation to the conditioning arena (context) for 6 min without tone or foot shock: mean % time spent freezing was about 5% in each group, in the absence of a Stressor effect (Supplementary Figure S1B). In accordance with the CSS-PALM model, at PAL testing (Figure 2B), whilst % time spent in CS-tone-related freezing increased monotonically across CS-US trials (Trials main effect: F_2, 148_ = 34.97, p < 0.001, η^2^ = 0.32), CSS mice acquired more CS freezing than did CON mice, as indicated by a Stressor × Trial interaction effect (F_2, 148_ = 5.30, p = 0.006, η^2^ = 0.07) and a Stressor main effect (F_1,74_ = 36.38, p < 0.001, η^2^ = 0.33). In CON mice and CSS mice, % time freezing at CS-US trials 5–6 was used to counterbalance mouse allocation to Compound groups (“Compound main effect”: F_2, 74_ < 1, p = 0.39, η^2^ = 0.02). Twenty-four hours after PAL testing, mice were administered VEH, PSY or ETH. After 3 days (72 h), mice underwent a PAM expression/extinction test, comprising 10 CS/context trials and nine ITIs. At PAM testing (Figure 2C), overall, mice maintained a consistent level of freezing across CS/context trials until the final trials (CS 9–10), when there was evidence for the onset of extinction across groups (Trials main effect: F_4, 296_ = 13.09, p < 0.001, η^2^ = 0.15). Again in accordance with the CSS-PALM model, CSS mice expressed more CS-tone/context-related freezing than did CON mice (Stressor main effect: F_1, 74_ = 7.12, p = 0.009, η^2^ = 0.09). Relative to mice that received VEH, mice that received psychedelic demonstrated increased CS/context PAM expression (Compound main effect: F_2, 74_ = 6.13, p = 0.003, η^2^ = 0.14; *post hoc* Sidak’s test: PSY vs. VEH, p = 0.041; ETH vs. VEH, p = 0.004). The Stressor × Compound interaction effect was non-significant (F_2, 74_ = 1.04, p = 0.36, η^2^ = 0.03). When PAL % time freezing in CS trials 5–6 was included as a covariate in ANCOVA, the Compound main effect on PAM CS/context freezing remained (F_2, 73_ = 5.61, p = 0.005, η^2^ = 0.13; *post hoc* Sidak’s test: PSY vs. VEH, p = 0.045; ETH vs. VEH, p = 0.007). When PAL % time freezing in ITIs 4–5 was included as a covariate–important given that by chance mice were not allocated evenly to compound groups with respect to ITI freezing (see below) and PAM testing was conducted in the same context as PAL conditioning, the Compound main effect on PAM CS/context freezing remained (F_2, 73_ = 5.47, p = 0.006, η^2^ = 0.13; *post hoc* Sidak’s test: PSY vs. VEH, p = 0.017; ETH vs. VEH, p = 0.018). The experiment was conducted with 2 littermates from each of 40 breeding pairs, with littermates assigned to the same treatment group. To investigate whether the observed effects of psychedelics on CS/context PAM freezing were attributable to the responses of certain litter pairs only, an additional mixed model analysis was conducted with fixed effects of Stressor, Compound and CS/context trials and a random effect of litter. Despite the reduction in statistical power due to the decrease in denominator degrees of freedom, almost all of the significant effects obtained in ANOVA using mouse as the unit of analysis, still pertained. Thus, in the absence of any interactions, there were main effects of Trials (F_4, 336_ = 9.60, p < 0.001), Stressor (F_1, 34_ = 4.72, p = 0.037) and Compound (F_2, 34_ = 4.06, p = 0.026); for the latter, in Sidak’s test there was no longer a significant increase in % time CS/context freezing in PSY littermate pairs vs. VEH littermate pairs (p = 0.138), whereas there was a significant increase in % time CS/context freezing in ETH littermate pairs vs. VEH littermate pairs (p = 0.030).

**FIGURE 2:**
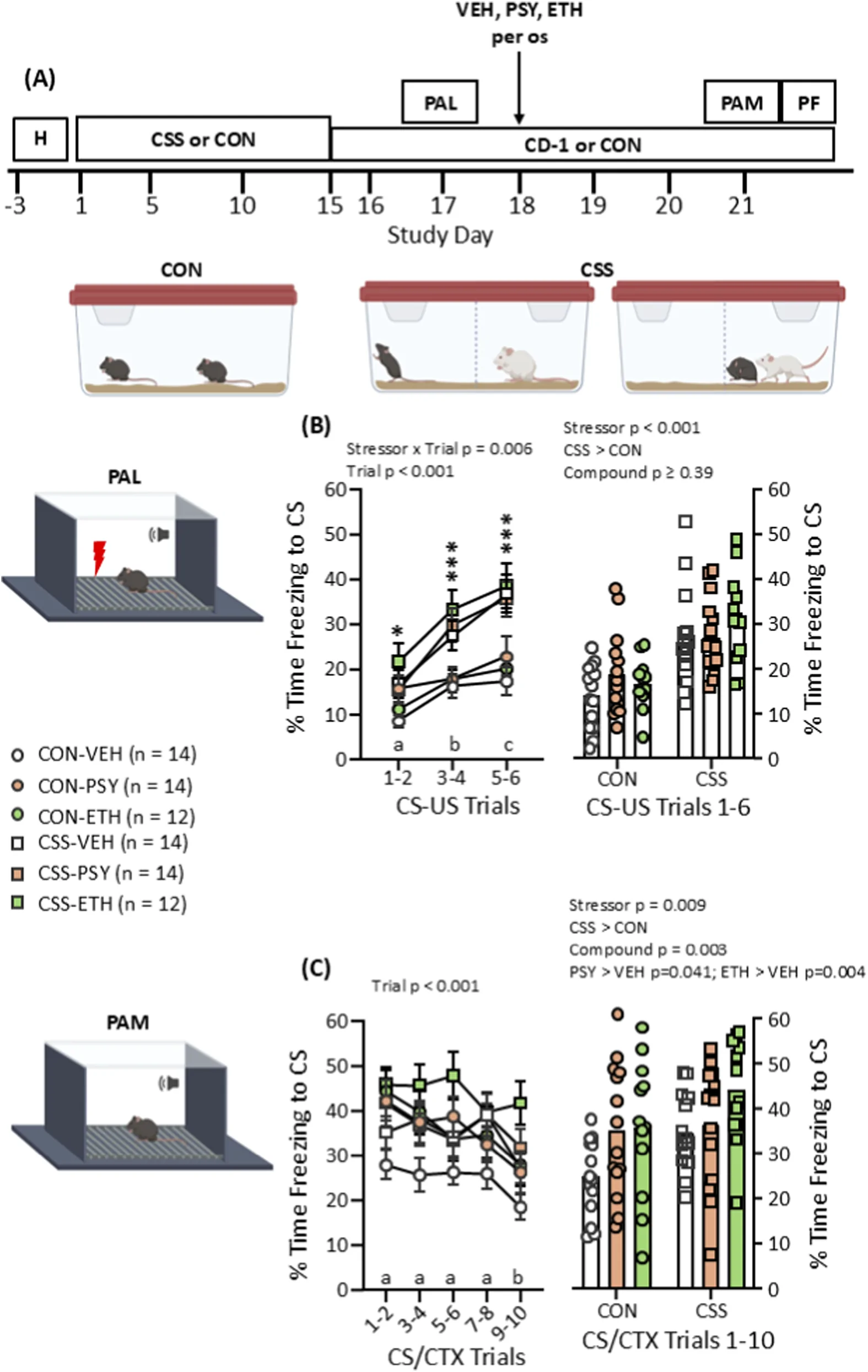
Sustained effects of psychedelics on Pavlovian aversion memory of tone-CS/context in control and CSS mice. Mice (n = 12–14 per stressor and compound or VEH) underwent control handling or chronic social stress followed by Pavlovian aversion learning, psychedelic administration and Pavlovian aversion memory testing. **(A)** Experimental design. H: handling; CSS or CON: 15-day chronic social stress or control handling; CD-1 or CON: CSS mice are caged with the same CD-1 mouse in separate compartments and without any attack until the end of the experiment; PAL: Pavlovian aversion learning; VEH, PSY, ETH per os: oral administration of PSY or ETH at 3 mg/kg in 5 mL/kg VEH; PAM: testing of Pavlovian aversion memory recall; PF: after PAM testing, deep anaesthesia with pentobarbital and perfusion-fixation. B, C. Left-hand graphs present Stressor-Compound mean ± SEM data for pairs of consecutive trials, and right-hand graphs present per mouse mean values and the overall mean for all pairs of trials. **(B)** Pavlovian aversion learning (PAL) measured as percent time spent in freezing behaviour during presentation of 6 trials of a tone-CS and footshock-US (analyzed as the mean of successive pairs of trials). Freezing increased across CS-US trials (Trials main effect: F2, 148 = 34.97, p < 0.001, η_2_ = 0.32, followed by Sidak’s test, with trials denoted by different letters, e.g., a vs. b, a vs. c, significantly different from each other at p < 0.05 or lower). CSS mice acquired more CS/context freezing than CON mice (Stressor × Trial interaction effect: F2, 148 = 5.30, p = 0.006, η_2_ = 0.07, followed by Sidak’s test at each CS-US trial pair; Stressor main effect: F1, 74 = 36.38, p < 0.001, η_2_ = 0.33). The counter-balanced allocation of mice to Compound is supported by the absence of a main or interaction effect of Compound. **(C)** Pavlovian aversion memory (PAM) recall of tone-CS/context measured as percent time spent freezing during presentation of 10 trials of a tone-CS (analyzed as the mean of successive pairs of trials) in the same context as for PAL. Across all Stressor-Compound groups, time spent freezing remained stable between trials 1–2 and 7–8 and then decreased at trials 9–10 (Trial main effect: F4, 296 = 13.09, p < 0.001, η_2_ = 0.15, followed by Sidak’s test, with trials denoted by different letters, i.e., a vs. b, significantly different from each other at p < 0.05 or lower, and trials denoted by the same letter, i.e., a vs. a, not significantly different from each other). CSS mice had excessive PAM of tone-CS/context relative to CON mice (Stressor main effect: F1, 74 = 7.12, p = 0.009, η_2_ = 0.09). Mice that received PSY or ETH had excessive PAM of tone-CS/context relative to VEH mice (Compound main effect: F2, 74 = 6.13, p = 0.003, η_2_ = 0.14; post hoc Sidak’s test: PSY vs. VEH, p = 0.041; ETH vs. VEH, p = 0.004). Post hoc, with a focus on early PAM expression, defined as CS/context trials 1–4, there was no Stressor main effect (p = 0.08) and mice that received PSY or ETH had excessive PAM of tone-CS/context relative to VEH mice (Compound main effect: F2, 74 = 7.11, p = 0.002, η_2_ = 0.16; post hoc Sidak’s test: PSY vs. VEH, p = 0.015; ETH vs. VEH, p = 0.002).

In the inter-trial intervals (ITIs) between CS/context-US trials (PAL) and CS/context trials (PAM), % time spent freezing is typically lower than during CS presentations; therefore, such freezing is regarded primarily as a specific measure of contextual learning and memory. In accordance with the CSS-PALM model, at PAL testing (Supplementary Figure S1B), whilst % time spent in CS-US ITI freezing increased monotonically across ITIs (ITI main effect: F_2, 148_ = 89.54, p < 0.001, η^2^ = 0.55), CSS mice acquired more ITI freezing than CON mice, as indicated by a Stressor × ITI interaction effect (F_2, 74_ = 4.81, p = 0.01, η^2^ = 0.11) and a Stressor main effect (F_1,74_ = 39.89, p < 0.001, η^2^ = 0.35). Whilst freezing at PAL CS/context-US trials 5–6 was used to counterbalance mouse allocation to compounds, for freezing at PAL ITI trials the counterbalancing was incomplete: there was a Stressor x “Compound” interaction effect (F_2, 74_ = 4.81, p = 0.011, η^2^ = 0.11) and Sidak’s test identified that ITI % time freezing was greater in CSS mice allocated to ETH than in CSS mice allocated to PSY (p = 0.011), with both groups similar to CSS mice allocated to VEH (Supplementary Figure S1B). At PAM testing (Supplementary Figure S1C), overall, mice maintained a consistent level of ITI freezing until ITIs 6–7, when there was evidence for the onset of extinction across groups (Trials main effect: F_4, 296_ = 6.99, p < 0.001, η^2^ = 0.09). Again in accordance with the CSS-PALM model, CSS mice expressed more ITI freezing than did CON mice (Stressor main effect: F_1, 74_ = 9.08, p = 0.004, η^2^ = 0.11). In contrast to the increased PAM freezing to CS/context in mice that received PSY or ETH, there was no evidence for an effect of PSY or ETH relative to VEH on PAM freezing during ITIs (Compound main effect: F_2, 74_ = 2.17, p = 0.12, η^2^ = 0.05). The p value for the Stressor × Compound interaction effect was less than 0.10 (F_2, 74_ = 2.41, p = 0.097, η^2^ = 0.06). When PAL % time freezing in ITIs 4–5 was included as a covariate in ANCOVA, the p value for the Stressor × Compound interaction effect increased markedly (F_2, 73_ < 1, p = 0.497, η^2^ = 0.02), suggesting a contribution of compound-group differences in PAL ITI freezing to PAM ITI freezing.

Further to the prespecified analysis of the 10 CS/context trials and nine ITIs, a *post hoc* analysis was conducted for the early period of memory expression, using the average per mouse CS/context % time freezing during trials 1–4 and the average per mouse ITI % time freezing during ITIs 1–3. For mean per mouse CS/context freezing at trials 1–4 (see Figure 2C left panel for group mean values at these trials), there was no significant difference between CSS and CON mice (e.g., Stressor main effect F_1, 74_ = 3.17, p = 0.08). There was a Compound main effect (F_2. 74_ = 7.11, p = 0.002, η^2^ = 0.16), with Sidak’s test identifying increased % time CS/context freezing in mice that received PSY (p = 0.015) or ETH (p = 0.002) compared with mice that received VEH. For mean per mouse ITI freezing at ITIs 1–3 (see Supplementary Figure S1C left panel for group mean values at these ITIs), CSS mice expressed more ITI freezing than did CON mice (Stressor main effect: F_1, 74_ = 4.21, p = 0.044, η^2^ = 0.05). Whilst there was a significant main effect of Compound (F_2, 74_ = 3.74, p = 0.028, η^2^ = 0.09), there were no significant *post hoc* pairwise differences (PSY vs. VEH, p = 0.074, ETH vs. VEH, p = 0.053).

For each Stressor-Compound group, the mice ranked 1 to 8 in terms of % time spent freezing to CS-tone/context in the PAM test (see Figure 2C) were studied in terms of PrL and AMYG levels of pre-synaptic vGluT1, post-synaptic Homer1, and vGluT1-Homer1 colocalization at 90 min/70 min after the onset/offset of PAM testing. Coronal sections including the target brain structures underwent immunofluorescence staining followed by confocal microscopy imaging of the region of interest in each structure and hemisphere (Figure 3A; Supplementary Figures S2–S4). For PrL layers II/III (Figure 3B), there was no effect of Stressor or Compound on these glutamate neuron synaptic proteins. For LA (Figure 3C), there were more vGluT1 puncta in CSS than CON mice, without a CSS effect on Homer1 and on vGluT1-Homer1 co-localization, and in the absence of a Compound effect on any parameter. For BA (Figure 3D), there was no effect of Stressor or Compound on the synaptic proteins investigated.

**FIGURE 3:**
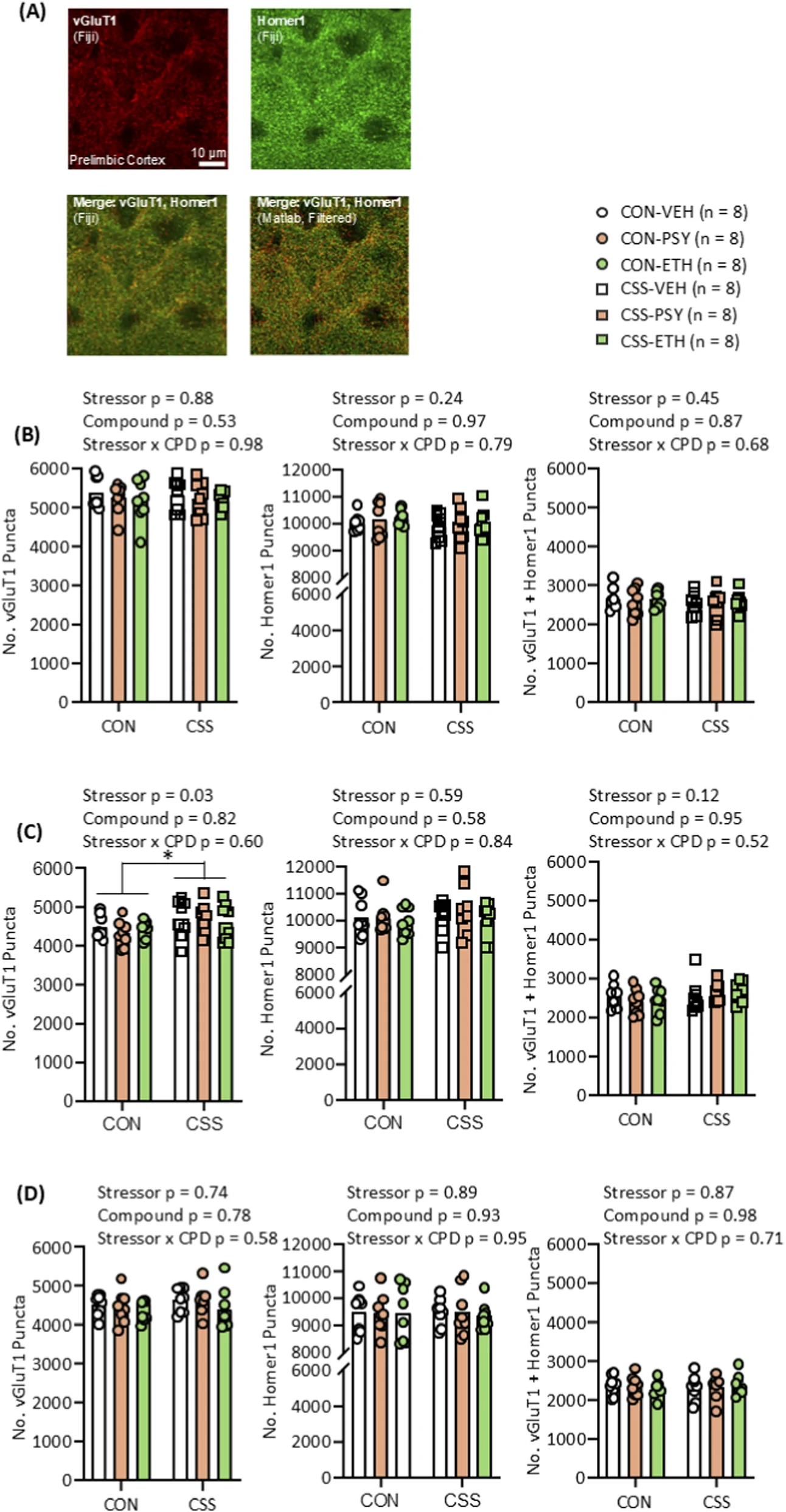
No effect of psychedelics on specific cortico-limbic glutamate neuron synaptic proteins in control or CSS mice post-PALM. Mice were perfused-fixed after PAM testing for immunofluorescence staining, imaging and quantification of glutamate synaptic proteins in PrL and AMYG ROIs in coronal tissue sections. Per mouse and structure/ROI, 2 sections and both hemispheres per section were assayed and quantified, and the mean puncta number was calculated and used for statistical analysis. **(A)** Representative micrographs of PrL showing immunofluorescence co-staining for vGluT1, used as pre-synaptic marker, and Homer1, used as post-synaptic marker. Micrographs: vGluT1 (red), Homer1 (green), merged vGluT1 and Homer1 (yellow). Scale bar = 10 µm. Brightness and contrast have been adjusted for display purposes. The lower-right image displays the visual output of the MATLAB script used for synaptic puncta detection and colocalization analysis for the images shown. In **B–D**, histograms display brain region ROI-specific number of puncta for vGluT1 (left), Homer1 (middle) and vGluT1-Homer1 puncta co-localization (right), according to Stressor and Compound, showing individual (n = 8 mice) and mean values. Statistical analysis was conducted using two-way ANOVA. **(B)** Prelimbic cortex. **(C)** Lateral amygdala. CSS mice had more vGluT1 puncta than CON mice (2-way ANOVA: F_1, 42_ = 5.24, p = 0.027). **(D)** Basal amygdala.

## 4 Discussion

Various hypotheses have been proposed to explain how serotonergic hallucinogens might contribute to improved treatment of stress-related psychiatric disorders. To investigate these, it is essential to complement human studies with animal model experiments. By necessity, the latter can contribute specifically–and potentially substantively–to understanding the involved subconscious and non-declarative psychological processes and underlying neurobiological circuits and mechanisms. In the present mouse study, we report that a single, 3 mg/kg oral dose of PSY or ETH led to acute increases in the blood and brain levels of their respective psychoactive metabolites, psilocin and ethocin, and to acute increases in head twitch responses. When the same dose was administered the day after Pavlovian aversion conditioning of tone and context to foot shock, either in control mice or mice that had undergone chronic social stress, and 3 days later tone-CS/context emotional memory testing was conducted, both PSY and ETH increased Pavlovian aversion memory expression. This sustained psychedelic effect was not associated with a change in glutamate neuron pre-synaptic vGluT1 or post-synaptic Homer1, or their colocalization, in either the prelimbic cortex or the lateral or basal amygdala, indicating that increased synaptogenesis involving these two proteins in these brain regions was not the basis of the observed stimulatory effect of psychedelics on aversive emotional memory.

Administering PSY or ETH orally to mice at 3 mg/kg is, based on allometric scaling (Nair and Jacob, 2016), equivalent to a dose of 0.24 mg/kg in human, and therefore equates to moderate-to-high in terms of the oral doses used in experimental-clinical psychiatric studies (e.g., (Kraehenmann et al., 2015; Carhart-Harris et al., 2016; Roseman et al., 2018; Barrett et al., 2020; von Rotz et al., 2023; Choi et al., 2024). It is noteworthy that a recent mouse study reported that 3.2 mg/kg PSY resulted in about 60% 5-HT_2A_ receptor occupancy in PFC (Maltby et al., 2026), which aligns with human findings that PSY at 0.20–0.27 mg/kg yielded 5-HT_2A_R occupancy of 60%–72% in healthy subjects (Madsen et al., 2019). In our pharmacokinetic experiment, for both PSY and ETH, blood plasma levels of their respective psychoactive metabolites, psilocin and ethocin, peaked at 15–30 min post-administration and declined monotonically thereafter, with concentrations predicted to be negligible beyond 120 min post-administration. For PSY, this pharmacokinetic profile and the absolute concentrations observed replicate our previous plasma findings (Thomann et al., 2024). In brain tissue–both cortical and subcortical–psilocin and ethocin levels were substantially higher at 60 min compared with 120 min post-administration. Extrapolating these findings to the HTR pharmacodynamic experiment, brain levels at 60 min provide approximate evidence for the exposure prevailing during HTR scoring across minutes 1–60 post-administration. For both compounds, mice displayed an increase in HTRs relative to the VEH condition. For PSY/psilocin, the absolute number of HTRs was at the lower end (7 in 15 min) of the reported range of 5–30 HTRs during 15–20 min after parenteral administration at 3 mg/kg (Halberstadt et al., 2011; Klein et al., 2020; Sherwood et al., 2020; Shao et al., 2021; Shahar et al., 2022; Maltby et al., 2026). Ethocybin/ethocin resulted in a higher number of HTRs (22 in 15 min) than was the case with PSY. This aligns with previous mouse findings that ethocin elicited more HTRs (58 and 36 in 30 min after 2 and 4 mg/kg i. p., respectively) than did psilocin (20 and 6 in 15 min after 1 and 3 mg/kg i. p., respectively) (Klein et al., 2020). This latter study also reported, for both compounds, a characteristic inverted-U curve for dose *versus* HTRs (Klein et al., 2020). Therefore, our and previous findings are consistent with ETH exerting a more potent acute and potentially hallucinogenic-like effect on mouse brain-behavior than does PSY. Whether this is related to differential engagement at 5-HT_2A_ and/or other 5-HT receptor subtypes, and/or to downstream effects on additional neurotransmitter systems, is unclear and will require further mechanistic investigations.

Rodent Pavlovian aversion learning and memory, so-called “fear conditioning”, is typically studied using auditory tone as CS and foot shock as US; this paradigm has been applied extensively to elucidate the neural circuitry of and cellular and molecular neurobiology underlying PALM, as a major example of Hebbian learning and synaptic plasticity (Johansen et al., 2011). It has also been applied to study the neural circuitry of emotional extinction learning and memory (Sierra-Mercado et al., 2011; Knapska et al., 2012). Previous rodent studies of effects of psychedelics on PAM expression/extinction have primarily used intra-peritoneal administration of a single dose of PSY (or structurally related 4-OH-DiPT) at 0.5–3.0 mg/kg. There are several reports of reduced freezing when PSY is administered shortly – 5–30 min–before memory testing (e.g., (Catlow et al., 2013; Hagsäter et al., 2021; Du et al., 2023; Kelly et al., 2024; Woodburn et al., 2024). It must be noted that direct, hallucinogenic-like effects will be on-going at this time point; furthermore, the presence of HTRs will physically confound the reliability of freezing time as a memory readout. With respect to potential longer-term effects, a recent study reported an absence of an effect of PSY at 2 mg/kg relative to vehicle in CS memory tests conducted directly before and at 24–48 h after administration (Lu et al., 2025). Psilocybin led to reduced CS freezing in repeated PAM expression/extinction tests: at 30 min, 1 day and 7 days post-administration, with the long-term effects specific to 1 mg/kg and absent at 0.5 or 2 mg/kg (Woodburn et al., 2024); and at 30 min, 1 day and 6 days post-administration at 2.5 mg/kg and not at lower doses (Du et al., 2023). Important differences between the designs of these studies and the current experiment, that could account for the differences in findings, include: In each of the other studies, the initial memory test was conducted a few minutes after PSY administration and therefore provided for a potential acute effect on reconsolidation and/or extinction that mediated reduced memory expression in the later memory tests. The CS memory tests were conducted in a different context to that used for conditioning, meaning that PSY effects on CS/context composite memory were assessed in the present study only. Whilst conducting CS testing in the conditioning context precludes separation of CS and contextual memories, it has the translational advantage of modeling the composite memories that are present and require treatments in stress-disorder patients. To date, only one previous study investigated PSY effects on PAM in mice that had undergone chronic stress: 3 mg/kg reduced freezing in a memory test at 30 min post-administration, but again the overlap with acute effects, including HTR induction, precludes direct comparison and necessitates caution (Razidlo et al., 2025).

That CSS leads to increases in both PAL and PAM in male mice, as demonstrated in the current as well as several previous studies, provides a robust model with which to study excessive generalized aversion responsiveness, its underlying neurobiology, and targets for its neuropharmacological reversal [e.g., (Azzinnari et al., 2014; Fuertig et al., 2016; Just et al., 2018; Adamcyzk et al., 2022; Sigrist et al., 2024; Poggi et al., 2025; Poggi et al., 2026)]. Administered at 1 day after PAL and 3 days before PAM, PSY and ETH had a sustained effect in terms of increasing aversion memory expression to the tone-CS/context. This psychedelic-induced increase in PAM freezing was present in the combined sample of CON mice and CSS mice. In an autoradiography study using the 5-HT_2A_R antagonist radioligand [^3^H]ketanserin, there was no CSS effect on 5-HT_2A_R binding in mPFC, where binding was the highest of all regions investigated; in amygdala, CSS mice had lower 5-HT_2A_R binding than did CON mice (Carneiro-Nascimento et al., 2021). The sustained psychedelic effect of PAM expression was specific to the tone CS/context and there was no effect of PSY or ETH compared with VEH on aversion memory expression during context only ITIs. In mice that received PSY or ETH 3 days previously, the increase in expression of the CS/context-conditioned response of freezing could well reflect increased retrieval of the emotional memory formed 4 days previously and 1 day prior to psychedelic administration. Another possibility is that psychedelic led to a sustained state of increased aversion sensitivity, such that the increased freezing reflected this rather than increased retrieval of an existing emotional memory. That this was not the case is suggested by the increase in freezing expression being specific to CS/context trials, whereas an increase in aversion sensitivity *per se* would be expected to be manifested as increased freezing to the context as well as to the CS + context. Psychedelic was administered 24 h after conditioning and therefore after the major cellular-molecular plasticity processes of consolidation (Schafe et al., 2001). Given that the experimental design included one memory retrieval-expression test it was not possible to study for psychedelic effects on reconsolidation. This effect of a single psychedelic administration in terms of a sustained increase in PAM expression was in the opposite direction to that of the SSRI escitalopram (3 mg/kg p. o.) when administered sub-chronically (11 days) between PAL and PAM: whilst without effect in CON mice, it normalized CS/context aversion memory expression in CSS mice (Poggi et al., 2025). This dichotomy of psychedelic and SSRI effects on PAM in mice has interesting parallels with their respective effects on human amygdala reactivity to fearful facial expressions in MDD: whereas SSRI attenuates amygdala fear-face reactivity (Godlewska et al., 2012; Godlewska and Harmer, 2021), PSY increases it (Roseman et al., 2018), with corresponding therapeutic interpretations of reduced aversion processing (Godlewska and Harmer, 2021) *versus* aversion confrontation/catharsis/resolution (Roseman et al., 2018), respectively.

Concerning the contributions of AMYG and mPFC to the neural circuitry of PALM, sensory thalamic and cortical glutamate neurons conveying CS and US signals are integrated at the synaptic level on post-synaptic pyramidal glutamate neurons of the LA (Johansen et al., 2011). These LA neurons project to central amygdala (CeA) GABA neurons, both directly and *via* BA (Duvarci and Pare, 2014). The LA/BA glutamate neurons also project to PrL, as well as PrL glutamate neurons projecting to LA/BA. Glutamate neurons of the LA/BA-to-PrL pathway fire in response to the auditory CS, and stimulation of this pathway increases expression of CS aversion memory (as freezing behavior) (Burgos-Robles et al., 2017). Glutamate neurons of the PrL-to-LA/BA pathway are activated during expression of CS aversion memory, and not during retrieval of extinction memory (Sierra-Mercado et al., 2011; Knapska et al., 2012). With respect to vGluT1 expression in pre-synaptic axon terminals: in LA this includes the terminals of glutamate sensory (thalamic, cortical) neurons and of PrL neurons; in BA it includes axon terminals of LA and PrL neurons; and in PrL it includes axon terminals of LA/BA neurons (Martineau et al., 2017). With respect to post-synaptic Homer1, this would include expression in synaptic spines on the dendrites of LA, BA and PrL neurons, including glutamate neurons expressing metabotropic glutamate receptor 1 (mGluR1) family glutamate receptors (Luo et al., 2012; Yoon et al., 2021) and iontropic glutamate receptors including AMPA and NMDA.

In the 8 out of 12 mice per Stressor x Compound group that were ranked highest in terms of their PAM CS/context freezing expression, whilst CSS was without effect on synaptic protein puncta in PrL and BA, it did lead to a sustained increase in vGluT1 puncta in LA, suggesting increased glutamate signaling by afferent neurons conveying CS and US signals to LA. In rats, chronic (21-day) restraint stress led to increased dendritic spines on glutamate neurons in LA/BA (Vyas et al., 2006). Both psychedelic compounds were without effect on vGluT1 and Homer1 in each of the brain structures examined at 3 days post-administration. In rat cortical cultures, acute application of the 5-HT_2A_R agonist psychedelics DOI and LSD led to increased vGluT1 puncta density, whilst the density of post-synaptic protein PSD-95 was unchanged (Ly et al., 2018). In mice, using repeated two-photon imaging, PSY at 1 mg/kg i. p. led to an increase in spine density in the mPFC including PrL; this occurred on the day of administration and persisted for 1 month (Shao et al., 2021). Given the various methods that have been applied to demonstrate synaptic effects of psychedelics, including *in vitro versus in vivo* neuropharmacology, and measurement of synaptic markers *versus* markers of dendritic spines, and given that the present *in vivo* study measured two of many synaptic proteins specifically, it would be entirely premature to conclude from the current findings that the demonstrated sustained behavioral effects of PSY and ETH occurred in the absence of any changes in glutamate neuron synapses and/or spines.

The current study includes several limitations. Firstly, because of the absence of female-female and male-female aggression, the CSS protocol can only be conducted in male mice, and therefore the entire study was confined to males. Indeed, most of the animal studies to date on the effects of psychedelics on mouse Pavlovian aversion behavior have been conducted with male mice. Second, when testing CS memory expression/extinction, we did so in the same context as used for conditioning, such that CS-context memory rather than pure CS memory was investigated. Nonetheless, the PAM-enhancing effects of PSY and ETH were specific to CS/context trials and were not present at inter-trial intervals, indicating that the CS was the major feature of the PAM memory impacted by psychedelic exposure. A test of current general anxiety state, such as the elevated plus maze, would be important in any similar, future study. A third limitation is that investigation of the neurobiological processes related to glutamate-neuron synaptic plasticity was limited to one pre- and one post-synaptic protein, and their co-localization, as markers of synaptic number and potential synaptogenesis. Other, potentially relevant synaptic proteins, such as pre-synaptic vGluT2 and post-synaptic PSD-95, were not measured, and we did not include any measure of spinogenesis. Given the current findings, future studies should investigate for sustained effects of PSY and ETH on spinogenesis, synaptogenesis and neurotrophic markers in glutamatergic and other types of neurons (Moliner et al., 2023). Finally, each of PSY and ETH were tested at a single dose rather than focusing on one psychedelic and testing it at two doses.

Therefore, in male mice a single per os administration of psychedelic compound, PSY or ETH, led to a sustained increase in Pavlovian aversion memory expression measured as increased time spent freezing during CS/context exposure. This is the first report of a sustained augmentation of aversion memory expression by psychedelics in mice. That psychedelics increased aversion memory is an effect opposite to that which would be taken as evidence for preclinical validation of a novel mechanism-of-action and, indeed, is opposite to that obtained with sub-chronic escitalopram (Poggi et al., 2025). However, extrapolating these findings to human patients of stress-related neuropsychiatric disorders in which aversion processing is excessive, it is a commonly expressed opinion that, for psychedelics to be effective (Choi et al., 2024), they need to be combined with psychotherapy [e.g., (Carhart-Harris et al., 2018; Grunder et al., 2024; Leone et al., 2024)]. In this regard, sustained increased retrieval of non-declarative aversion memories, including of otherwise suppressed memories, rendering them more accessible to subsequent conscious expression, processing and extinction, could constitute a desirable neuro-psycho-pharmacological state (Wolff et al., 2020; Passie et al., 2022; Zeifman et al., 2023).

## Data Availability

The raw data supporting the conclusions of this article will be made available by the authors, without undue reservation.
