# Supplementary material for: Acute psilocybin or ethocybin leads to a sustained increase in Pavlovian aversion memory in male mice
Source: Front Pharmacol. 2026 Sep 22;17:1918182. doi: 10.3389/fphar.2026.1918182 (PMC13639876; doi:10.3389/fphar.2026.1918182)
Supplement: Supplementary file 1 [file DataSheet1.pdf]

Supplementary Information

Table S1. Mass spectrometry parameters.

	Mass transition Q1→Q3 (*m/z*)	Declustering potential (V)	Entrance potential (V)	Collision energy (V)	Collision cell exit potential (V)	Calibration range plasma (ng/mL)	Calibration range brain homogenate (ng/mL)
Psilocin	205.1→58.1	61	10	11	12	0.5–1000	1–1000
Ethocin	233.2→86.1	61	10	6	36	2.5–1000	1–1000

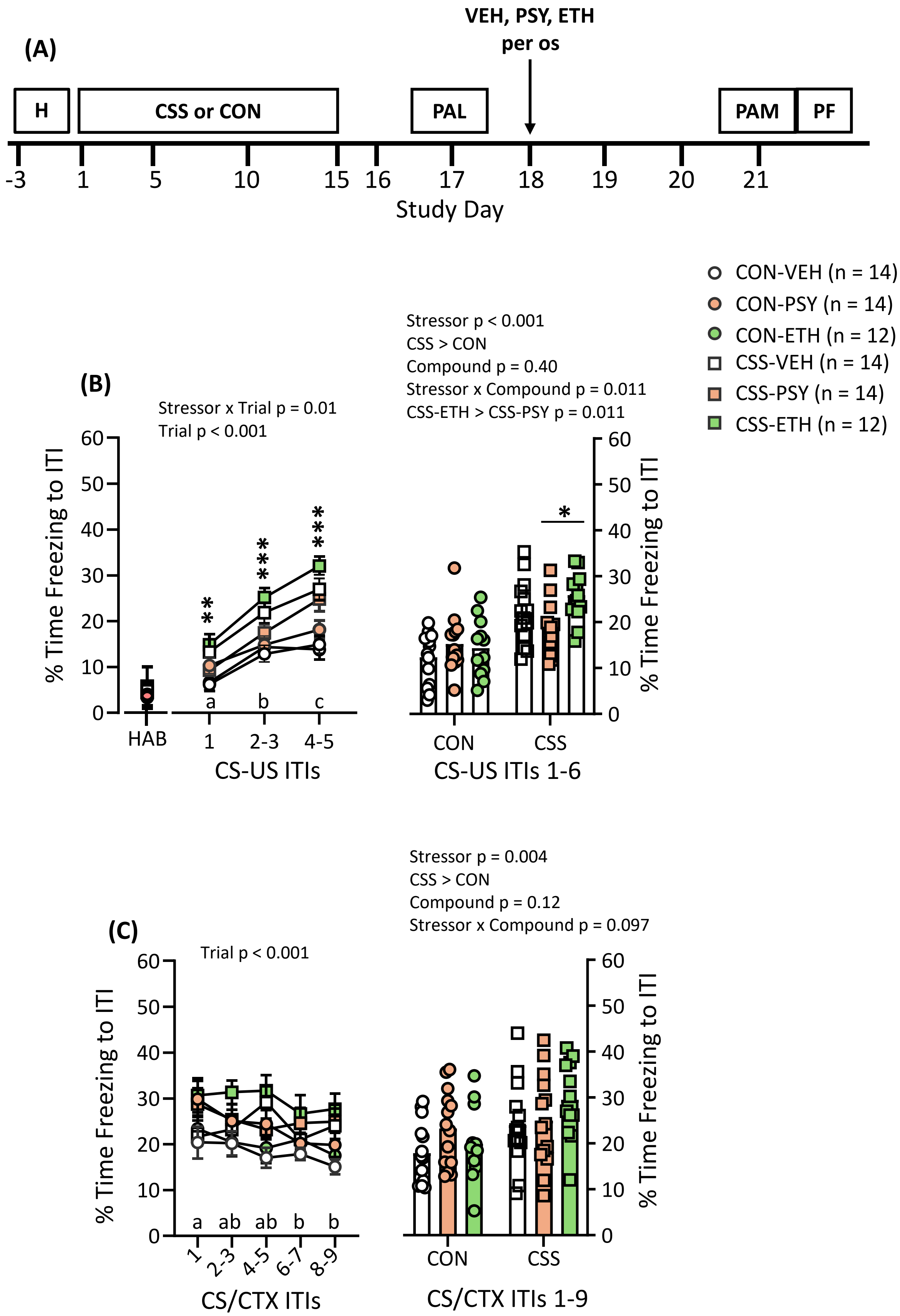
(A)
VEH, PSY, ETH
per os
H
CSS or CON
PAL
PAM
PF
-3
1
5
10
15
16
17
18
19
20
21
Study Day
CON-VEH (n = 14)
CON-PSY (n = 14)
CON-ETH (n = 12)
CSS-VEH (n = 14)
CSS-PSY (n = 14)
CSS-ETH (n = 12)
(B)
Stressor x Trial p = 0.01
Trial p < 0.001
Stressor p < 0.001
CSS > CON
Compound p = 0.40
Stressor x Compound p = 0.011
CSS-ETH > CSS-PSY p = 0.011
% Time Freezing to ITI
0
10
20
30
40
50
60
a
b
c
HAB
1
2-3
4-5
CS-US ITIs
CON
CSS
CS-US ITIs 1-6
(C)
Trial p < 0.001
Stressor p = 0.004
CSS > CON
Compound p = 0.12
Stressor x Compound p = 0.097
% Time Freezing to ITI
a
ab
ab
b
b
1
2-3
4-5
6-7
8-9
CS/CTX ITIs
CON
CSS
CS/CTX ITIs 1-9

Figure S1. No effect of psychedelics on Pavlovian aversion memory of context in control and CSS mice. Mice (n = 12-14 per stressor and compound or VEH) underwent control handling or chronic social stress followed by Pavlovian aversion learning, psychedelic administration and Pavlovian aversion memory testing. **(A)** Experimental design. H: handling; CSS or CON: 15-day chronic social stress or control handling; CD-1 or CON: CSS mice are caged with the same CD-1 mouse in separate compartments and without any attack until the end of the experiment; PAL: Pavlovian aversion learning; VEH, PSY, ETH per os: oral administration of PSY or ETH at 3 mg/kg in 5 mL/kg VEH; PAM: testing of Pavlovian aversion memory recall; PF: at 90 min post-PAM onset, deep anesthesia with pentobarbital and perfusion-fixation. **B, C.** Left-hand graphs present Stressor-Compound mean ± SEM data for pairs of consecutive inter-trial intervals (ITIs), and right-hand graphs present per mouse mean values and the overall mean for all pairs of ITIs. **(B)** Percent time spent freezing during habituation (HAB, no effect of Stressor or Compound: $p \geq 0.18$) and, directly thereafter, contextual Pavlovian aversion learning (PAL) measured as percent time spent in freezing behavior during 5 ITIs between 6 CS-US trials of aversion conditioning. Freezing increased across ITIs (ITI main effect: $F_{2, 148} = 89.54$, $p < 0.001$, $\eta^2 = 0.55$, followed by Sidak's test, with trials denoted by different letters, e.g. a vs b, a vs c, significantly different from each other at $p<0.05$ or lower); CSS mice acquired more ITI freezing than CON mice (Stressor x Trial interaction effect: $F_{2, 74} = 4.81$, $p = 0.01$, $\eta^2 = 0.11$, followed by Sidak's test at each ITI pair; Stressor main effect: $F_{1,74} = 39.89$, $p < 0.001$, $\eta^2 = 0.35$). A Stressor x Compound interaction effect with respect to ITI freezing ($F_{2, 74} = 4.81$, $p = 0.011$, $\eta^2 = 0.11$) indicated a non-counterbalanced allocation of CSS mice to Compound and Sidak's test identified that ITI % time freezing was greater in CSS mice allocated to ETH than in CSS mice allocated to PSY ($p = 0.011$), with both groups similar to VEH. **(C)** Pavlovian aversion memory (PAM) recall of context measured as percent time spent freezing during 9 ITIs between CS trials. Overall, time spent freezing during ITIs was lower at ITIs 8-9 versus ITI 1 (Trial main effect: $F_{4, 296} = 6.99$, $p < 0.001$, $\eta^2 = 0.09$, followed by Sidak's test, with trials denoted by different letters, i.e. a vs b, significantly different from each other at $p<0.05$ or lower, and trials denoted by the same letter, e.g. a vs ab, b vs b not significantly different from each other). CSS mice expressed more PAM ITI freezing than CON mice (Stressor main effect: $F_{1, 74} = 9.08$, $p = 0.004$, $\eta^2 = 0.11$). In contrast to the increased PAM freezing to CS/context in mice that received PSY or ETH, there was no evidence for an effect of PSY or ETH relative to VEH on PAM freezing during ITIs (Compound main effect: $F_{2, 74} = 2.17$, $p = 0.12$, $\eta^2 = 0.05$). Post hoc, with a focus was on early PAM expression, defined as ITIs 1-3, CSS mice expressed more ITI freezing than did CON mice (Stressor main effect: $F_{1, 74} = 4.21$, $p = 0.044$, $\eta^2 = 0.05$). There was a significant main effect of Compound ($F_{2, 74} = 3.74$, $p = 0.028$, $\eta^2 = 0.09$), in the absence of any significant post hoc pairwise differences (PSY vs VEH, $p = 0.074$, ETH vs VEH, $p = 0.053$).

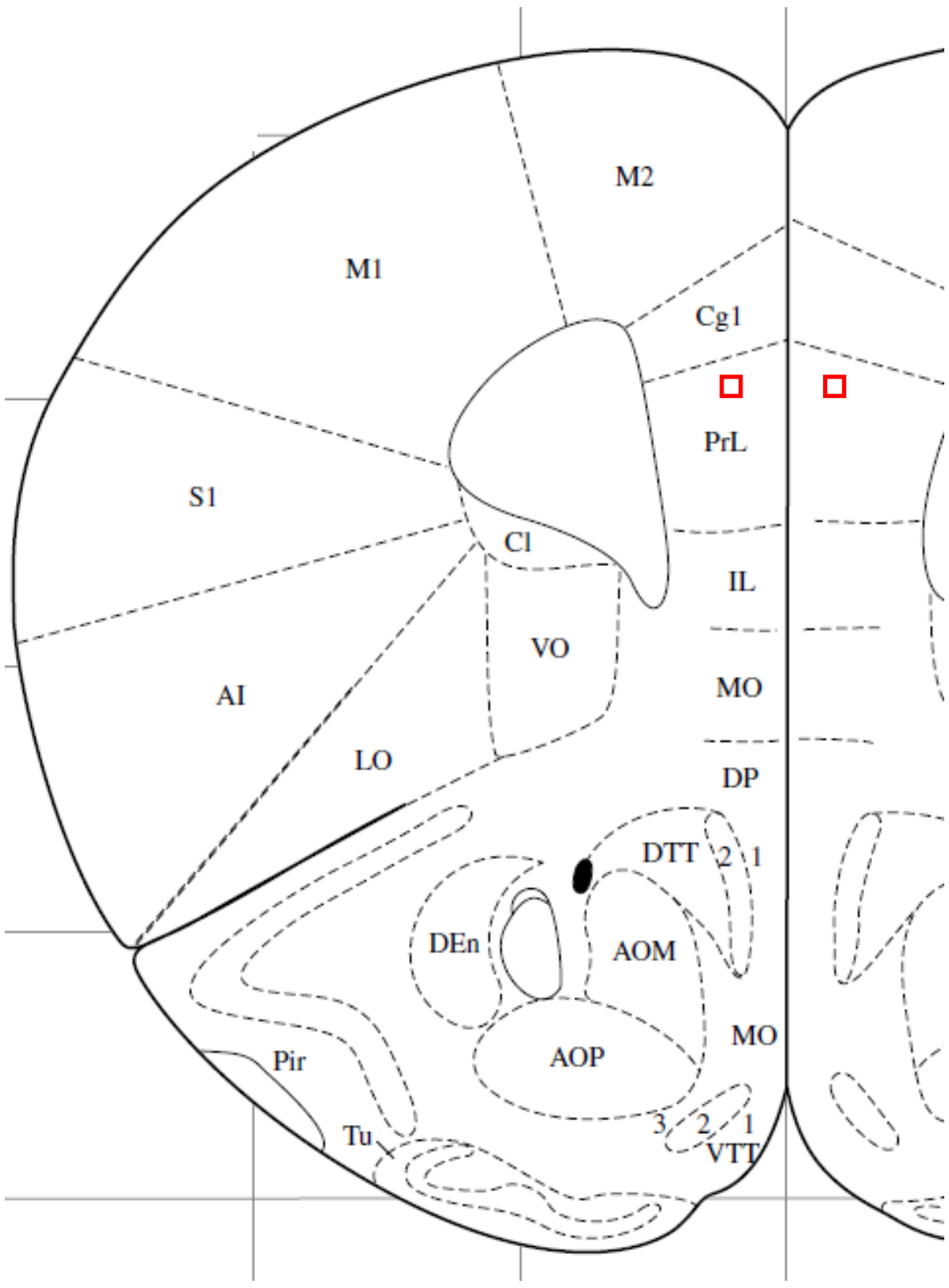

Figure S2. Coronal-section image from the Mouse Brain Atlas (Paxinos & Franklin, 2001), containing the PrL (bregma 2.0 mm). The red squares indicate the locations and the approximate size of the ROIs at image acquisition.

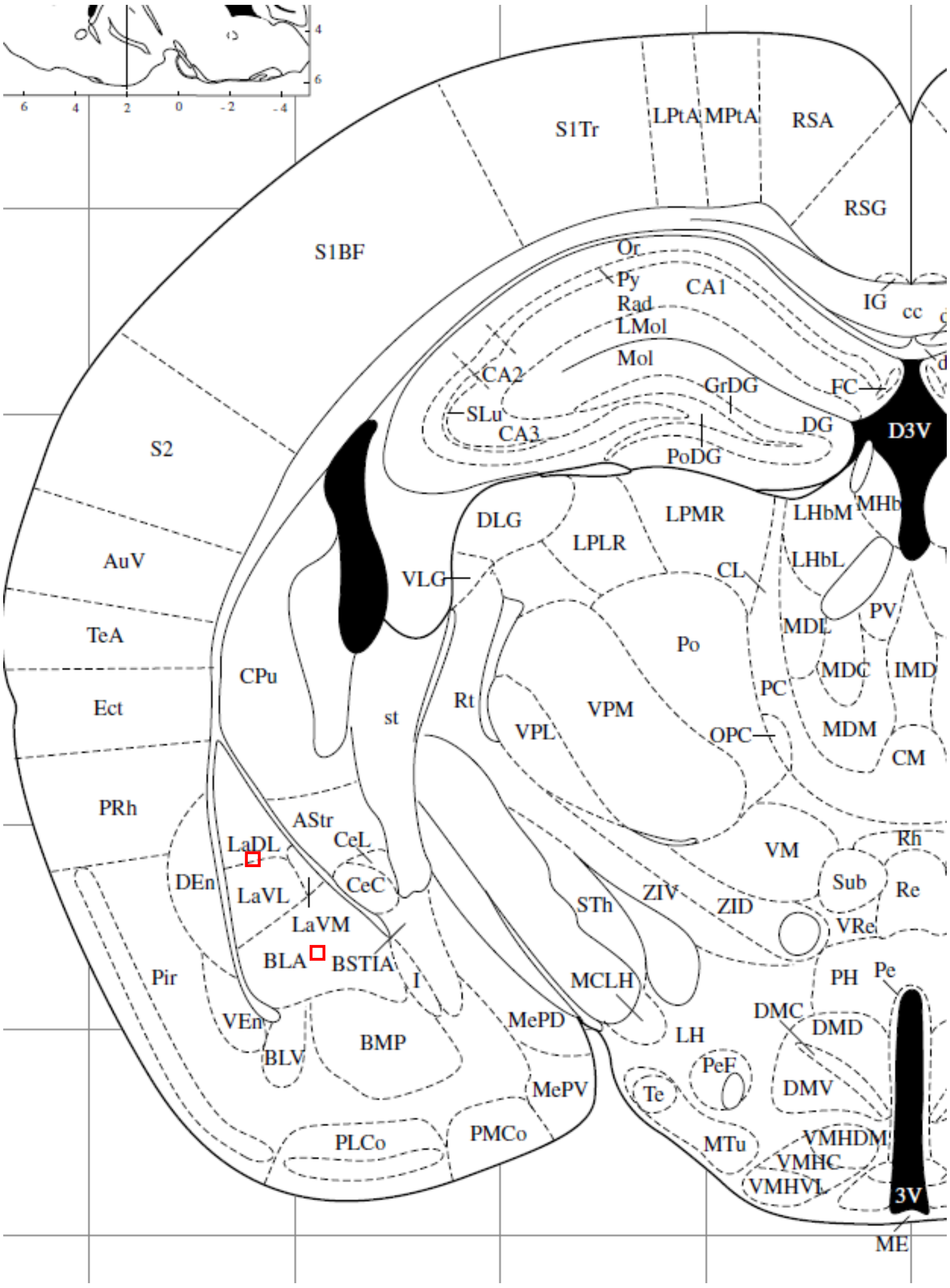

Figure S3. Coronal-section image from the Mouse Brain Atlas (Paxinos & Franklin, 2001), containing the AMYG (LA and BA, bregma -1.8 mm). The red squares indicate the locations and the approximate size of the ROIs at image acquisition.

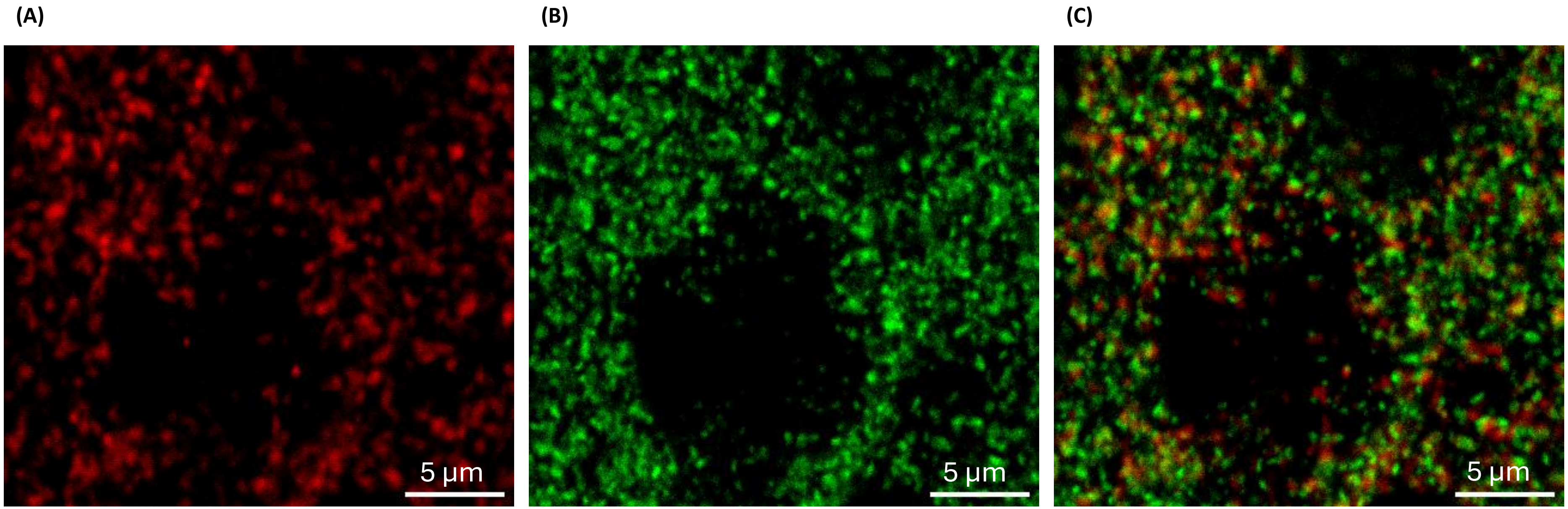

Figure S4. Synaptic protein immunofluorescence. Representative micrographs of prelimbic cortex showing immunofluorescence co-staining for (A) vGluT1, used as pre-synaptic marker, (B) Homer1, used as post-synaptic marker, (C) merged vGluT1 and Homer1 indicating the presence of co-localized (adjacent) vGluT1-Homer1 puncta, which were interpreted and quantified as synapses by the MATLAB script . Scale bar = 5 µm. Brightness and contrast have been adjusted for display purposes.
